# DFGLM-TCM: an integrated knowledge-and experience-driven large language model system for Traditional Chinese Medicine practice

**DOI:** 10.1186/s13020-026-01512-y

**Published:** 2026-08-28

**Authors:** Ruikang Zhong, Dianna Liu, Yuewen Dai, Zexing Li, Zilin Song, Zhixing Li, Le Zhang, Siyi Chen, Yisha Su, Yue Sun, Wenzheng Zhang, Pengfei Jia, Min Jiang, Lei Gao, Kaiwen Hu

**Affiliations:** 1 Dongfang Hospital, Beijing University of Chinese Medicine, Beijing, China; 2 Beijing University of Chinese Medicine, Beijing, China; 3 Beijing Zhipu Huazhang Technology Co., Ltd., Beijing, China; 4 Beijing Fengtai Integrated Traditional Chinese and Western Medicine Hospital, Beijing, China

**Keywords:** Traditional Chinese Medicine, Large language model, DFGLM-TCM, Integrated clinical support system, Practitioner-specific experience

## Abstract

Traditional Chinese Medicine (TCM) clinical practice depends on both codified theoretical knowledge and practitioner-specific experience, which differ in their data sources, reasoning patterns, and scope of generalization. We developed DFGLM-TCM, a modular large language model-based service system that separately models these two knowledge types through task-oriented components and coordinates them within a unified multi-task architecture. The knowledge-oriented component integrated a curated 22-GB TCM corpus containing approximately 3 million structured entries, a manually validated knowledge graph with more than 200,000 entities, and retrieval-augmented generation for literature retrieval and general TCM question answering. The experience-oriented component was trained using approximately 5000 authentic outpatient records from a senior TCM practitioner and 2000 expert-reviewed augmented cases to provide practitioner-specific diagnostic and prescription references. Through standardized interfaces and role-based access, the system supports knowledge retrieval, question answering, structured consultation, and prescription reference for clinicians, patients, and students. In an expert-rated evaluation of 100 TCM knowledge questions, DFGLM-TCM achieved the highest descriptive mean score among the evaluated models (4.48 ± 0.76). In 454 independent pulmonary-nodule cases, the experience-oriented component achieved a prescription-consistency score of 8.95 ± 0.63, exceeding that of the model additionally trained with general TCM knowledge (7.52 ± 0.44). A one-month assessment involving 35 physicians at three primary-care institutions suggested favorable short-term usability and acceptance. These findings highlight the value of separating general TCM knowledge from practitioner-specific experience while coordinating task-oriented training and multi-task services within a unified system, and support further evaluation of DFGLM-TCM as an auxiliary reference tool in broader clinical settings.

**Supplementary Information:**

The online version contains supplementary material available at https://doi.org/10.1186/s13020-026-01512-y.

## Introduction

Digital and intelligent transformation has become a core component of modern healthcare systems [1, 2], driven by increasing clinical complexity and the growing demand for scalable, high-quality medical services [3]. Large language models (LLMs) have demonstrated substantial potential in medical question answering [4], clinical document processing [5, 6], and decision support [7]. However, translating these technological advances into routine clinical practice remains challenging, particularly in knowledge-intensive and experience-driven domains [8] that require multi-step, context-sensitive, and human-supervised workflows [9, 10].

Traditional Chinese Medicine (TCM) represents such a domain. TCM practice integrates the Four Examinations, syndrome differentiation, treatment-principle formulation, and individualized prescription design. Clinical decisions depend not only on codified knowledge from classical texts, textbooks, and guidelines, but also on practitioners’ accumulated experience and context-dependent interpretation of patient information [11]. These characteristics make TCM difficult to support using conventional clinical guidelines or one-size-fits-all intelligent tools [12], thereby creating distinct requirements for the representation and computational modeling of general TCM knowledge and clinical experience.

Senior TCM practitioners play a central role in the accumulation and transmission of clinical expertise. Through decades of practice, they develop relatively stable diagnostic and therapeutic patterns that are continually refined through clinical experience [13]. Much of this expertise remains tacit and is difficult to formalize through textbooks or standardized guidelines. Although apprenticeship and outpatient teaching are important mechanisms for knowledge transfer, their scalability and accessibility are limited by differences in learning opportunities, individual interpretation, and the distribution of expert resources [14]. As a result, a persistent gap has emerged between medical education and clinical practice, particularly in primary healthcare settings. Computational approaches that preserve both general TCM knowledge and practitioner-specific experience may help bridge this gap by providing complementary support for education, knowledge transfer, and clinician-facing reference support.

Recent advances in LLMs and related techniques, including retrieval-augmented generation, knowledge graphs, and multi-agent systems, have created new opportunities for improving knowledge grounding, reasoning, and interaction in medical applications [15–18]. TCM-oriented LLMs have evolved from general domain-knowledge injection for focused tasks toward broader integration of clinical data, multi-task capabilities, and expert-informed alignment. MedChatZH applies continual pre-training on TCM literature followed by instruction fine-tuning for TCM consultation and medical question answering [19], while TCM-KLLaMA incorporates symptom, tongue, and pulse information through a TCM knowledge graph to support herbal prescription generation [20]. BianCang further extends domain adaptation through staged knowledge injection and task alignment, covering syndrome differentiation, disease diagnosis, medical dialogue, and related tasks [21]. Zhongjing introduces expert-ranked feedback to improve multi-turn consultation and proactive inquiry [22], whereas Tianyi progressively integrates classical texts, expert treatises, clinical records, and knowledge graphs to support broad TCM knowledge and clinical tasks [23]. In parallel, the field has expanded from relatively focused tasks toward multi-task applications, with increasing emphasis on clinically oriented evaluation and real-world validation. However, general TCM knowledge and expert-derived clinical information are commonly integrated within a unified model or sequential training pipeline. The explicit separation of broad knowledge grounding from the modeling of an individual practitioner’s diagnostic and prescribing patterns as independently optimized components remains comparatively underexplored. Given their differences in data sources, generalization scope, optimization objectives, and evaluation criteria, a modular framework that coordinates these components across multiple service tasks and clinical workflows warrants further investigation.

Accordingly, we developed DFGLM-TCM, a modular LLM-based service system that separates general TCM knowledge from practitioner-specific clinical experience while coordinating them within a unified architecture. The knowledge-oriented component combines a curated TCM corpus and knowledge graph with retrieval-augmented generation to support literature retrieval and general TCM question answering, whereas the experience-oriented component is fine-tuned using clinical records from one senior TCM practitioner to model practitioner-specific diagnostic and prescribing patterns. Through standardized service interfaces, the two components support multiple functions, including knowledge retrieval, question answering, structured consultation, and practitioner-specific prescription reference, with role-oriented access for clinicians, patients, and students. The system is intended to provide auxiliary reference support rather than replace clinical decision-making. We evaluated the knowledge-oriented component using expert-curated TCM questions, assessed the experience-oriented component through prescription-consistency comparisons in pulmonary-nodule cases, and examined system feasibility and usability in primary-care settings. This study investigated the potential of task-specific modeling and system-level integration to coordinate codified TCM knowledge and experience-intensive clinical support across educational and clinical workflows.

## Methods

### Overall system design and study framework

This study investigated the feasibility of deploying a large language model-based digital intelligence service system to support TCM education and clinical practice. DFGLM-TCM was designed as a modular system that explicitly separates two modeling targets: general TCM knowledge and practitioner-specific clinical experience. This separation was based on their differences in data sources, intended outputs, scope of generalization, optimization objectives, and evaluation criteria. The knowledge-oriented component was designed to emphasize broad knowledge coverage, retrievability, and external knowledge grounding, whereas the experience-oriented component was designed to model the practitioner’s diagnostic and prescribing patterns using clinical-record mining and reasoning-chain supervision. The two components were trained and optimized independently and coordinated through standardized interfaces at the service layer.

We constructed a domain-specific TCM corpus and knowledge graph and developed two task-aligned LLM components. The knowledge-oriented component supports literature retrieval and general TCM question answering, while the experience-oriented component provides practitioner-specific diagnostic and prescription references. These components, together with the underlying data resources, were integrated into a unified service architecture supporting knowledge retrieval, medical question answering, structured multi-round consultation, and practitioner-specific prescription reference. Structured consultation workflows and role-oriented interfaces were implemented at the service and application layers to support clinicians, patients, and TCM students according to their respective needs.

**Fig. 1:**
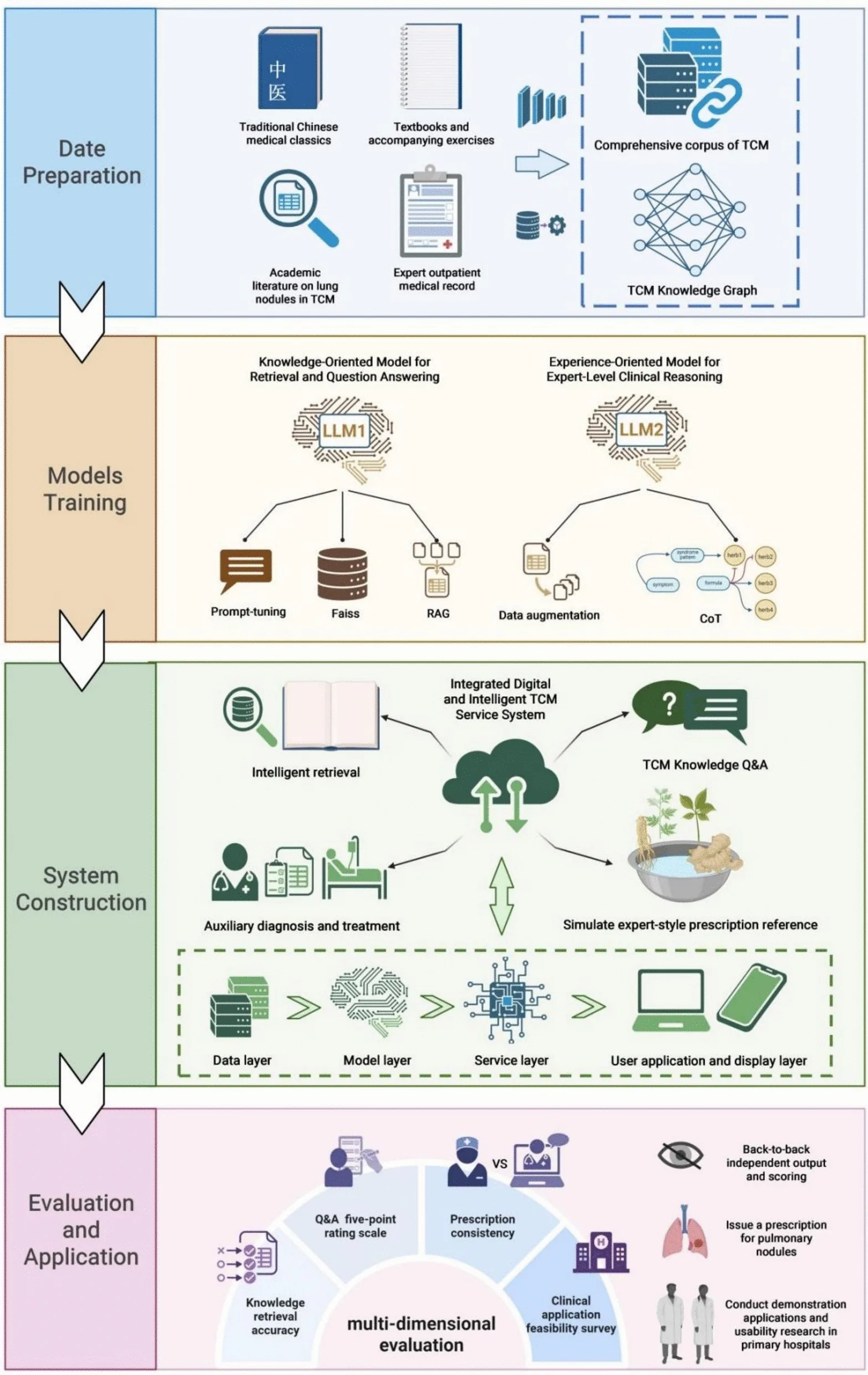
Overall System Architecture and Research Framework of DFGLM-TCM. The framework spans multi-source TCM data curation, task-oriented model adaptation, service-level integration, component-specific evaluation, and preliminary primary-care feasibility assessment. The knowledge-oriented and experience-oriented components coordinate knowledge retrieval, question answering, structured consultation, and practitioner-specific prescription reference through a unified service architecture

The system was intended to provide auxiliary information and reference support rather than replace clinical decision-making. The study evaluated the knowledge-oriented and experience-oriented components using task-specific assessment methods and further examined the feasibility and usability of their coordinated implementation in primary-care settings. Figure 1 illustrates the overall study framework, including data curation, independent model adaptation, service-level integration, task-specific evaluation, and primary-care implementation assessment.

### Data sources and knowledge resources

To support specialized training and system operation, a comprehensive TCM corpus and a structured knowledge graph [24] were constructed. The corpus integrated data from multiple sources, including classical Chinese medical texts, modern textbooks, exercise collections, peer-reviewed scientific literature, clinical guidelines, and related educational materials. Collectively, these materials covered foundational TCM theories, diagnostic principles, syndrome differentiation, herbal medicine, prescriptions, and clinically relevant domain knowledge. To support the modular design of DFGLM-TCM, general TCM knowledge resources and practitioner-specific clinical data were organized as separate data streams. During source-document selection, duplicate files, repeated editions, and redundant copies of the same source materials were manually reviewed and excluded before corpus ingestion.

To process multi-source, heterogeneous textual data, a standardized workflow was employed, comprising text extraction, text cleansing, structured processing, and corpus construction and optimization. Optical Character Recognition (OCR) technology was applied for text extraction, followed by text cleansing to remove formatting artifacts and encoding inconsistencies [25]. Regular expressions were applied to identify and extract structured elements, such as titles, chapters, and paragraphs, thereby reconstructing hierarchical document structures for subsequent processing. The processed text was imported into a MongoDB [26] database via an automated Extract-Transform-Load (ETL) pipeline, enabling batch ingestion, version control, and iterative optimization. Quality control was ensured through manual sampling and expert review, with particular attention to OCR-generated content. The corpus schema and classification definitions were continuously refined to support downstream knowledge structuring, graph construction, and domain-specific model training.

The knowledge graph was developed with reference to the fifth edition of Chinese Medical Dictionary, with a primary focus on case-based classical TCM texts [27]. It emphasizes the modeling of intrinsic relationships among disease, symptom, prescription, and medicinal entities, defining eight entity types and their corresponding semantic relations represented as triples. Five representative texts were first selected: Treatise on Febrile Diseases, Synopsis of the Golden Chamber, Treatise on the Spleen and Stomach, Integrating Chinese and Western Medicine, and Shennong’s Classic of Materia Medica. These texts are foundational works in TCM education, with a strong emphasis on clinical case analysis and herbal interpretation. Complete manual annotation was performed on these five classical texts, which served as gold-standard training data.

Low-Rank Adaptation (LoRA) was applied to fine-tune ChatGLM3-6B (Table 1), adapting the model for entity recognition and semantic relationship identification in classical Chinese medical texts [28]. Subsequently, prompt templates were designed and employed to enable batch extraction of entities and semantic relations from the target classical Chinese medical texts. Each prompt template comprised text input, extraction instructions, output format constraints, entity-relation scope definitions, and illustrative examples. Following model-based extraction, medical experts reviewed and corrected the results, and the refined annotations were fed back to support iterative model optimization. The finalized annotations were uniformly structured in JSON format. Following the relational logic of the TCM knowledge graph, entities and relations were imported into the Neo4j graph database, thereby constructing the finalized TCM knowledge graph. The resulting knowledge graph served as a structured resource for knowledge retrieval, grounding, and visualization within the knowledge-oriented component.

**Table 1:** Fine-tuning hyperparameter configuration

Hyperparameters	Values
Batch size	128
Max epoch	10
Learning rate	5e−4
LoRA rank	8
LoRA alpha	16
LoRA dropout	0.05
LoRA target modules	q_proj, v_proj

### Domain-specific model training and vertical adaptation

To align large language models with the knowledge system and clinical reasoning paradigms of TCM, domain adaptation was performed using a preconstructed comprehensive knowledge base and structured clinical data. Unlike monolithic or unified training approaches, differentiated technical pathways were adopted for distinct functional modules within the system: knowledge-oriented models for information retrieval and question answering, and experience-oriented models for clinical reasoning and prescription reference. This design reflects the inherently heterogeneous nature of TCM practice, in which factual knowledge acquisition and experience-driven decision-making impose distinct requirements on data representation, training strategies, and evaluation metrics.

Intelligent knowledge retrieval and general TCM knowledge question-answering tasks, which are primarily knowledge-driven, require broad semantic coverage, robust language understanding, and effective integration with external knowledge sources. Prior research in medical NLP has demonstrated that large pre-trained models, when combined with RAG and instruction-level adaptation, can deliver reliable, factually grounded responses without requiring extensive task-specific parameter tuning [29, 30]. To this end, prompt tuning combined with knowledge base integration was employed to align the model with TCM terminological systems and interpretive conventions, while leveraging external knowledge bases to provide authoritative contextual support during reasoning.

Specifically, based on the structured TCM corpus and knowledge graph, textual representations were indexed using Facebook AI Similarity Search (Faiss) to support efficient semantic retrieval [31]. Discrete knowledge elements were extracted, integrated, and linked to form a structured knowledge base. The knowledge base was incorporated into a RAG framework, with supervised fine-tuning performed using the ChatGLM-130B pre-trained model. During inference, the system first retrieves relevant knowledge elements from the repository, which are then provided as contextual input to the large language model, enabling the generation of more accurate and comprehensive responses. Through this mechanism, the system achieves integrated knowledge retrieval and question-answering capabilities.

In contrast, simulating the diagnostic and therapeutic reasoning of renowned senior TCM practitioners requires deep contextual understanding across multi-step clinical encounters, personalized interpretation of symptom information, and coherent, experience-driven treatment logic. Accordingly, GLM4-32B was adopted, a model characterized by a relatively compact architecture and enhanced semantic understanding and reasoning capabilities. Full-parameter fine-tuning [32] was performed using curated clinical case data, in combination with data augmentation [33] and chain-of-thought-based reasoning supervision [34]. This approach allows the model to internalize experience-based reasoning patterns, while avoiding excessive reliance on pre-training priors optimized for broad knowledge coverage.

For data preparation, the experience-oriented model was trained using a combination of real-world expert outpatient records and augmented clinical cases generated through structured reasoning chains. The expert source was Professor Kaiwen Hu from Dongfang Hospital, Beijing University of Chinese Medicine, a nationally recognized TCM practitioner and Qihuang Scholar with over three decades of clinical experience in herbal medicine for tumor prevention and treatment. Symptom-pattern-prescription-herb reasoning trajectories were first extracted from the expert’s outpatient records collected over the past three years. These trajectories capture the prescribing logic and reasoning structures commonly employed by experienced TCM practitioners. Subsequently, the expert team reviewed and refined these reasoning chains to ensure internal consistency. Based on the reviewed reasoning structures, symptom patterns, syndrome interpretations, and treatment principles were systematically reorganized to generate additional synthetic clinical cases under controlled conditions. Approximately 2000 augmented cases [35] were generated and iteratively reviewed by the expert team. These augmented cases were combined with 5000 authentic outpatient records to form the final training dataset. This strategy was intended to expand coverage of expert decision-making patterns while reducing the risk of overfitting associated with limited clinical data.

Initial training utilized the full pre-trained weights of GLM4-32B, with all parameters explicitly set as trainable. Subsequently, end-to-end full-parameter supervised fine-tuning was conducted using the aforementioned dataset. Within the dataset, diagnostic information was explicitly encoded as symptom-syndrome-prescription-herb reasoning chains derived from expert clinical practice. To achieve stable optimization under memory constraints, gradient accumulation and learning rate scheduling were employed during training. Large-scale full-parameter updates were efficiently executed using distributed training acceleration and mixed-precision computation. Twenty percent of the dataset was allocated as a validation set for continuous monitoring of model performance. Training loss was recorded at fixed intervals (every five optimization steps) to track convergence behavior, and the model was periodically evaluated on the validation set (every 100 steps) to assess generalization performance on unseen cases. Regular checkpoints were saved, and the final model was selected based on validation performance, serving as the system’s experience-guided clinical reasoning engine.

To investigate the impact of training data composition on expert-oriented prescription modeling, we conducted a controlled comparative experiment using two progressively trained models based on the same GLM4-32B backbone and identical optimization settings. The only experimental variable was the composition of the supervised fine-tuning data. In the first training configuration, the model was fine-tuned exclusively on a meticulously curated expert-derived dataset as mentioned earlier. This dataset preserves clinical diagnostic logic, prescription structures, and expert-specific medication patterns, thereby directly aligning with prescription evaluation and generation tasks. In the second-stage configuration, the converged model from the first stage was further fine-tuned using incremental structured general TCM knowledge extracted from the comprehensive TCM corpus (16,021 samples). This incremental dataset covers diverse knowledge categories within the TCM corpus and was introduced to evaluate whether general TCM knowledge augmentation can further improve prescription-related performance. Both models were trained under identical full-parameter fine-tuning configurations, including learning rate scheduling, batch size, optimization strategy, and evaluation intervals, ensuring that any observed performance differences could be examined primarily in relation to training-data composition.

### System architecture and multi-module integration

DFGLM-TCM adopts a layered architecture to enable coordinated integration and interaction among data resources, domain-adapted models, service modules, and user-facing applications, thereby supporting scalable deployment in educational and clinical support scenarios. The architecture comprises five layers: the data layer, model layer, service layer, user application layer, and presentation layer.

The data layer, which serves as the foundational infrastructure of the system, integrates heterogeneous TCM data resources. These resources include curated TCM corpora, case-centered classical medical texts, expert consultation records, and structured TCM knowledge graphs. Stored in structured and semi-structured formats, these data are exposed through unified access interfaces. The data layer supports downstream tasks such as knowledge retrieval, reasoning support, and model training, while ensuring data consistency across service modules.

The model layer integrates two vertically adapted large language models, each performing distinct yet complementary functions. The knowledge-oriented model supports intelligent retrieval and general TCM knowledge question answering, whereas the experience-oriented model provides practitioner-specific diagnostic and prescription references. Each model operates independently while collaborating through standardized interfaces, enabling flexible deployment across service tasks without functional role overlap.

The service layer encapsulates core system capabilities into modular services, including intelligent literature retrieval, TCM knowledge question answering, structured consultation workflows, and practitioner-specific prescription reference generation. Each service module communicates with the model and data layers through standardized application programming interfaces implemented using FastAPI [36], supporting modular composition, reuse, and extensibility. This layer also employs workflow-level constraint mechanisms to ensure that model outputs remain aligned with predefined functional scopes.

The user application layer provides role-based access control for different user groups, such as clinicians, patients, and TCM students. Access permissions and available functions are dynamically determined by user roles, ensuring appropriate and controlled utilization of system capabilities. User inputs are collected through structured interfaces or service calls, enabling controlled interactions and standardized evaluation across application scenarios.

The presentation layer delivers system outputs through user interface (UI) and user experience (UX) design. This layer is decoupled from core computation and business logic, enabling flexible deployment across devices and application scenarios.

Overall, the system is organized into a hierarchical structure that supports multi-model collaboration, multi-service integration, and role-oriented applications within a unified framework. This layered design provides the technical basis for component-specific evaluation and preliminary implementation in educational and clinical-support settings.

### Evaluation of the knowledge-oriented component

For the evaluation of knowledge question-answering performance, two TCM experts curated a test set of 100 questions (Supplementary Table 1) and provided standardized reference answers. The test questions covered a broad range of TCM domains, including classical theories, syndrome concepts, treatment principles, classical texts, and clinical specialties such as internal medicine, surgery, gynecology, and pediatrics. The questions and reference answers were independently selected and compiled by the expert panel from designated standardized TCM textbooks. Although these textbooks were included among the source materials of the general TCM corpus, the curated questions and answers were not directly used as supervised training samples. Accordingly, this evaluation was intended to assess knowledge retrieval, information integration, and answer generation within the predefined TCM knowledge domain. A 5-point Likert scale-based scoring rubric [37] (Table 2) was applied to rate the quality of the generated responses. All responses were independently scored by three associate chief TCM physicians. Before evaluation, model identifiers and other identifying information were removed. Responses from different models were randomly reordered and assigned anonymized codes, thereby blinding the evaluators to model identity. The evaluators scored the responses independently and had no access to one another’s ratings. When the difference between the highest and lowest scores for the same response was 2 points or greater, the response was reviewed and adjudicated by a TCM physician holding the professional title of chief physician.

**Table 2:** Knowledge Q&A scoring rules

1 point	Off-topic
2 point	Able to answer questions, but contains obvious errors or fails to use relevant theories from TCM
3 point	Able to answer questions from a TCM perspective without obvious errors, but not comprehensive (covers less than 60% of knowledge points)
4 point	Able to answer questions from a TCM perspective, with no obvious errors and relatively comprehensive coverage (covering ≥60% and <80% of key points)
5 point	Accurate responses from a TCM perspective, with no obvious errors, comprehensive and professional (covering ≥80% of key points)

For comparative evaluation, the same 100 questions were submitted to GPT-4o, Gemini 3 Pro, Llama 3.3-70B, DeepSeek-V3.2, Baichuan-M3, HuatuoGPT-II, and DFGLM-TCM using a standardized procedure. All models were accessed on January 19, 2026. Each question in Supplementary Table 1 was copied verbatim and submitted as the sole user input in a separate new conversation using a zero-shot direct-question prompting strategy. The first complete response generated by each model was retained, and no response was regenerated or selectively excluded.

Temperature and top-p were not manually specified during the original evaluation. The default decoding configurations of the respective platforms were used, and the exact parameter values could not be retrospectively retrieved from the interaction interfaces. No external documents or manually configured retrieval resources were provided to the comparison models. DFGLM-TCM used its built-in Faiss-based retrieval-augmented generation workflow, with the same system configuration applied to all 100 questions. The same three TCM physicians evaluated the responses generated by all models using identical scoring criteria.

### Prescription-consistency evaluation of the experience-oriented component

**Fig. 2:**
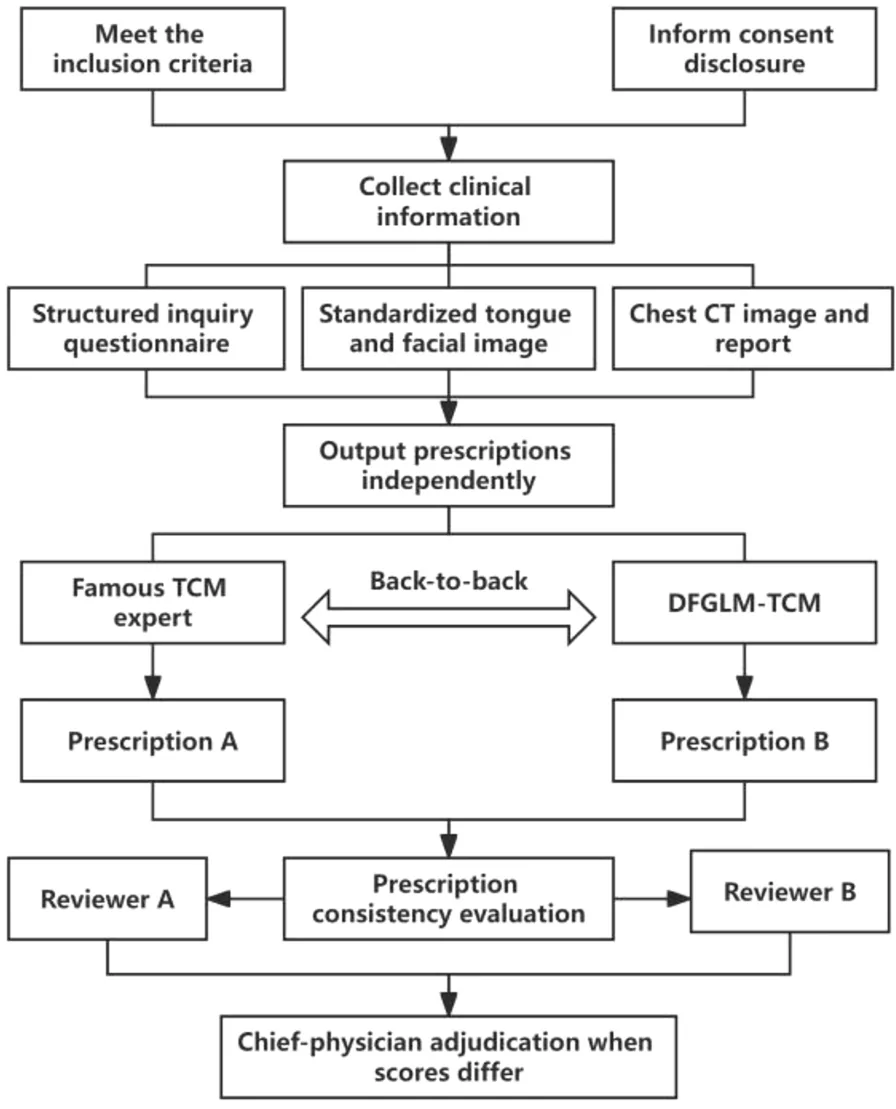
Workflow for the prospective prescription-consistency evaluation

To assess the extent to which the experience-oriented component reproduced practitioner-specific prescribing patterns, a prospective prescription-consistency evaluation was conducted using prospectively collected pulmonary-nodule cases (Fig. 2). The study protocol was approved by the Ethics Committee of Beijing University of Chinese Medicine Dongfang Hospital (Approval No.: JDF-IRB-2023032202) prior to implementation. All patient data were anonymized to ensure participant privacy and data protection. A total of 454 pulmonary-nodule cases were prospectively collected exclusively for evaluation. Inclusion criteria included: (i) CT-confirmed pulmonary nodules; (ii) ability to provide complete medical history; (iii) provision of informed consent; (iv) absence of comorbid conditions affecting the chief complaint or prescription; and (v) no psychiatric disorders, dementia, or other cognitive impairments. Data collection comprised structured questionnaires, standardized tongue and face images captured using a dedicated imaging device, chest CT scans, and corresponding radiology reports. These prospective evaluation cases were independent of the retrospective outpatient records collected from Professor Kaiwen Hu over the preceding three years. No case-level overlap existed between the 454 evaluation cases and either the authentic expert records or the augmented cases generated from the extracted diagnostic and therapeutic reasoning chains. None of the prospective evaluation cases was used for model training, validation, data augmentation, or model selection.

For each case, the collected information was independently provided to both the system and the senior TCM practitioner. The system and the practitioner generated prescriptions independently without access to each other’s outputs. Before evaluation, the prescriptions were anonymized and their sources were concealed from the evaluators. The prescriptions were compared using a comprehensive scoring rubric (Supplementary Table 2) developed by five senior TCM clinicians. The rubric assessed prescription consistency, coverage of practitioner-specific medication preferences, predefined prescription-level safety items, and generation efficiency. The evaluation emphasized consistency with practitioner-specific prescribing patterns rather than exact prescription matching. Among these criteria, only compatibility consistency required expert assessment. Two associate chief TCM physicians independently scored this item, and disagreements were resolved by review from a chief physician, with the adjudicated score used as the final result. Other criteria were calculated according to predefined computational rules. Inter-rater reliability for compatibility consistency was evaluated using ICC(A,2). The resulting scores were interpreted as measures of prescription-pattern consistency within this expert-specific and disease-specific setting, rather than clinical efficacy.

### Primary-care feasibility and usability assessment

To assess the preliminary feasibility and usability of DFGLM-TCM in primary-care settings, a one-month deployment demonstration was conducted at three designated primary-care hospitals. The system was integrated into routine clinical support activities as a demonstration platform, enabling primary-care physicians to access its core functionalities. During the demonstration, the system was used within a predefined workflow for pulmonary-nodule consultations. Primary-care physicians interacted with the system through a standardized interface and were encouraged to explore its functionality in routine outpatient scenarios. System-generated information and prescription references were provided solely as auxiliary references, and final diagnostic and therapeutic decisions remained the responsibility of the participating physicians. Role-based access permissions were applied to restrict system functions according to user roles, and data included in the research analysis were anonymized.

Following system deployment, a questionnaire survey [38] was conducted among participating primary-care physicians to evaluate user experience with the system. The survey focused on key dimensions relevant to primary-care practice, including: (i) usefulness for daily diagnostic and therapeutic reference; (ii) compatibility with existing clinical workflows; (iii) clarity and interpretability of system outputs; (iv) perceived usefulness of AI-generated prescription references; and (v) willingness to use the system and suggestions for improvement. A 5-point Likert scale was used to assess user perceptions. This assessment focused on short-term usability, acceptability, perceived usefulness, and workflow compatibility, and it was not intended to evaluate clinical efficacy or patient outcomes.

## Results

### Construction results of the TCM corpus and knowledge graph

**Fig. 3:**
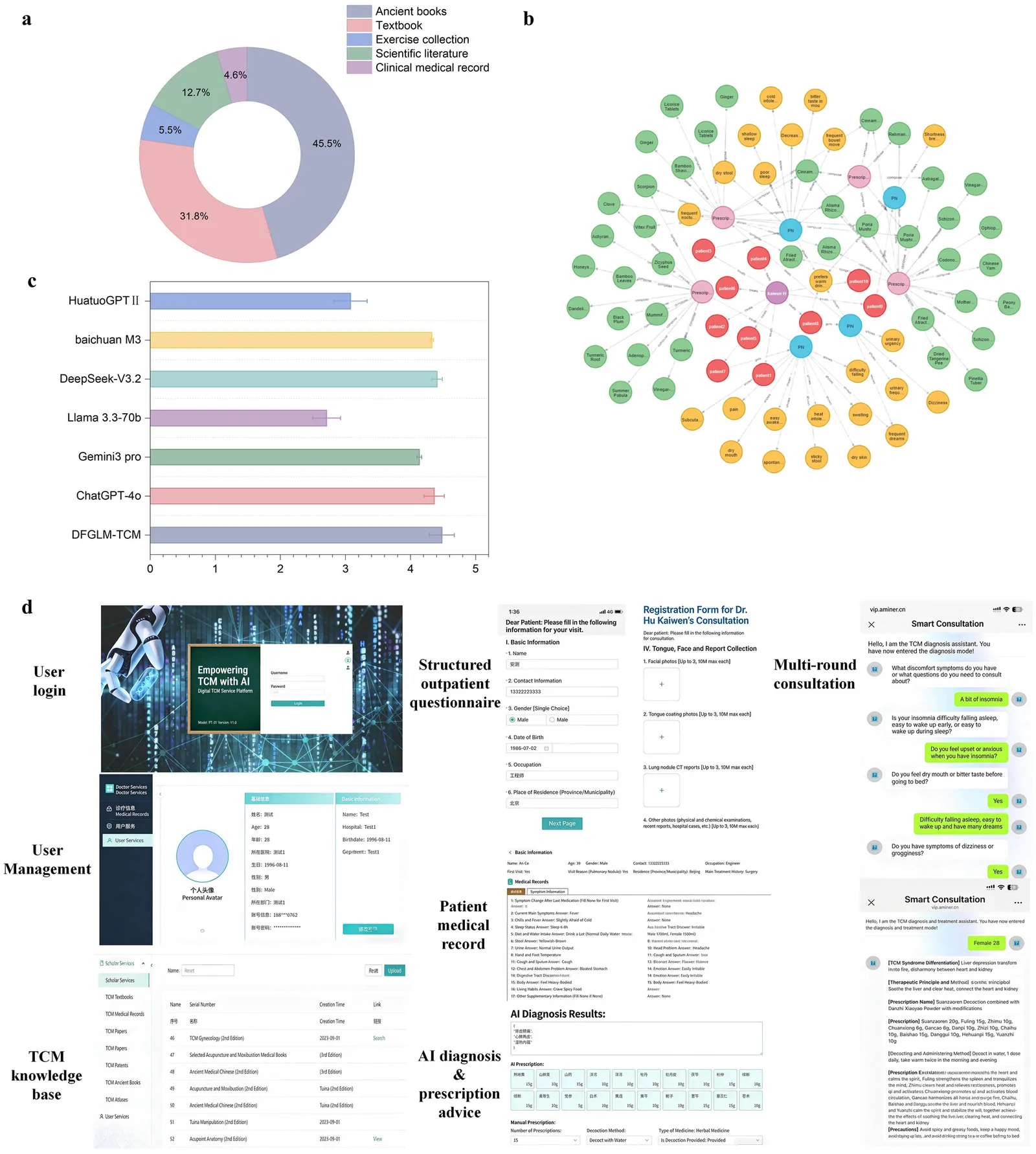
Data Resources, Knowledge Graph, Knowledge Q&A Evaluation, and System Interfaces of DFGLM-TCM. **a** Source categories of the overall data resources used in DFGLM-TCM, including general TCM knowledge resources and practitioner-specific clinical records. These resources were maintained as separate data streams for the knowledge-oriented and experience-oriented components, respectively. **b** Example of the TCM knowledge graph, showcasing structured entities and relationships extracted from classical texts and clinical knowledge, with interactive visualization. **c** Knowledge Q&A module evaluation using a five-point Likert scale to assess relevance, accuracy, and interpretability, with comparisons to other large models. **d** Examples of the DFGLM-TCM front-end interfaces

Through the established processing workflow, this study constructed a large-scale, heterogeneous TCM knowledge corpus. The source collection totaled approximately 22 GB, comprising approximately 17 GB of classical TCM texts and standardized textbooks, 4 GB of peer-reviewed journal articles and clinical guidelines, and 1 GB of examination questions and related educational materials. Following data cleaning and hierarchical structuring, the final usable corpus comprised approximately 3 million structured text entries. The resulting corpus covered major TCM domains, including fundamental theories, diagnostic principles, syndrome differentiation, herbal medicine, formulas, and clinical applications (Fig. 3a).

To better align with clinical needs, we constructed a medical case-centric knowledge graph for traditional Chinese medicine. Through entity and relationship extraction from 107 representative case-based classical texts, followed by manual validation, the resulting knowledge graph comprised over 200,000 entities spanning eight major categories, including symptoms, syndromes, treatment principles, formulas, herbal medicines, and their interrelationships (Table 3). The model achieved an entity recognition and relation extraction accuracy of 86.7%. After manual verification, the system produced high-quality structured outputs, resulting in a complete knowledge graph implemented in the Neo4j graph database. Both the TCM corpus and the knowledge graph were integrated into the system’s front-end visualization interface, enabling interactive exploration of TCM knowledge (Fig. 3b).

**Table 3:** Entity-relationship types

Entity	Relationship types
Disease	Disease → Manifestation → Symptom
Symptoms	Tongue Appearance → Composition → Symptoms
Tongue appearance	Pulse Pattern → Composition → Symptoms
Pulse pattern	Chinese Herbal Medicine → Composition → Prescription
Formula	Formula → Treatment → Disease
Herbal	Formula → Treatment → Symptoms
Dosage instructions	Formula → Usage → Dosage Instructions
Formula analysis	Formula → Contains → Formula Analysis

### Knowledge question-answering performance evaluation

To evaluate TCM knowledge retrieval and question-answering performance, DFGLM-TCM was compared with representative general-purpose and medical-domain large language models, including GPT-4o, Gemini 3 Pro, Llama 3.3-70B, DeepSeek-V3.2, Baichuan-M3, and HuatuoGPT-II. The evaluation results are summarized in Fig. 3c and Table 4.

**Table 4:** Knowledge Q&A Scores

	Evaluator 1	Evaluator 2	Evaluator 3	Overall score	ICC(A,3), 95% CI	Within-one-point agreement, %
DFGLM-TCM	4.60 ± 0.89	4.58 ± 0.79	4.26 ± 1.01	4.48 ± 0.76	0.789 (0.699–0.854)	83.0
GPT-4o	4.30 ± 0.93	4.25 ± 0.98	4.54 ± 0.78	4.36 ± 0.80	0.855 (0.794–0.900)	91.0
Gemini 3 Pro	3.84 ± 0.90	4.15 ± 0.78	4.16 ± 0.71	4.05 ± 0.70	0.822 (0.740–0.879)	92.0
Llama 3.3-70B	2.47 ± 0.77	2.78 ± 0.95	2.89 ± 0.97	2.71 ± 0.76	0.780 (0.682–0.849)	82.0
DeepSeek-V3.2	4.35 ± 0.83	4.50 ± 0.83	4.37 ± 0.82	4.41 ± 0.69	0.778 (0.691–0.844)	91.0
Baichuan-M3	4.09 ± 0.83	4.30 ± 0.77	4.34 ± 0.61	4.24 ± 0.61	0.750 (0.650–0.825)	90.0
HuatuoGPT-II	2.86 ± 0.85	3.01 ± 1.10	3.37 ± 0.97	3.08 ± 0.86	0.826 (0.732–0.885)	85.0
All model-question responses					0.888 (0.870–0.904)	87.7

DFGLM-TCM achieved the highest descriptive mean score, with an average composite score of 4.48 ± 0.76 across all questions. DeepSeek-V3.2 (4.41 ± 0.69) and GPT-4o (4.36 ± 0.80) achieved comparable scores, followed by Baichuan-M3 (4.24 ± 0.61) and Gemini 3 Pro (4.05 ± 0.70). Llama 3.3-70B (2.71 ± 0.76) and HuatuoGPT-II (3.08 ± 0.86) received lower scores in this evaluation. Their lower-rated responses were more frequently judged to contain factual or theoretical inaccuracies, particularly for questions requiring integrated understanding of classical theory and clinical reasoning. The model-specific ICC(A,3) values ranged from 0.750 to 0.855, and the within-one-point agreement rates ranged from 82.0% to 92.0%. Across all model-question responses, the overall ICC(A,3) was 0.888 (95% CI, 0.870–0.904), with a within-one-point agreement rate of 87.7%.

### Prescription-consistency evaluation results

A prospective prescription-consistency evaluation was conducted to assess the extent to which the experience-oriented component reproduced practitioner-specific prescribing patterns. A total of 454 patients with confirmed pulmonary nodules were included exclusively in the evaluation cohort. Baseline characteristics are summarized in Supplementary Table 3; women accounted for 55.95% of the cohort, and the mean age was 52.18 ± 15.08 years. Cases were distributed across Lung-RADS 2022 [39] categories 2 (46.04%), 3 (23.13%), and 4 (29.74%); classifications were unavailable for five cases (1.10%).

The system-generated prescriptions showed a high level of consistency with the expert prescriptions, with an overall consistency score of 8.95 ± 0.63. The mean similarity-component score was 6.62 ± 0.61. The prescriptions contained a median of 24 herbs (range, 20–24), meeting the predefined prescription-size criterion and receiving a score of 1 point. The mean score for disease-related herbs was 0.94 ± 0.23, and the compatibility-consistency score was 3.17 ± 0.56. The prescriptions achieved greater than 90% coverage of the predefined set of herbs commonly used in the practitioner’s outpatient practice, corresponding to a score of 1.5 points. All prescriptions received full scores for the two predefined safety items concerning herb incompatibility and toxic-herb usage. The mean prescription-generation time was 23.54 ± 5.28 s. For the manually assessed compatibility consistency item, the two evaluators demonstrated excellent agreement. The ICC(A,2) was 0.968 (95% CI, 0.943–0.987), with a mean inter-rater difference of 0.020 ± 0.140 (Table 5).

**Table 5:** Detailed scoring of prescription consistency

	Practitioner-specific data only	General TCM knowledge + practitioner-specific data
Similarity	6.62 ± 0.61	4.65 ± 0.43
Prescription size	1.00 ± 0.00	1.00 ± 0.00
Specialized medication for specific diseases	0.94 ± 0.23	0.93 ± 0.26
Compatibility consistency	3.17 ± 0.56	1.75 ± 0.33
Habitual medication	1.50 ± 0.00	0.98 ± 0.11
Safety	2.00 ± 0.00	2.00 ± 0.05
Incompatibility of drugs in a prescription	1.0 ± 0.00	1.00 ± 0.05
Use of toxic drugs	1.00 ± 0.00	1.00 ± 0.00
Prescription output speed	0.33 ± 0.17	0.86 ± 0.15
Total score	8.95 ± 0.63	7.52 ± 0.44

To assess the influence of incorporating general TCM knowledge, a comparative model was trained using an incremental dataset combining practitioner-specific records with 16,021 general TCM knowledge samples from the curated corpus. On the same evaluation cohort, the incrementally trained model achieved a similarity subtotal of 4.65 ± 0.43, including scores of 1.00 ± 0.00 for prescription size, 0.93 ± 0.26 for disease-specific herbs, 1.75 ± 0.33 for compatibility consistency, and 0.98 ± 0.11 for practitioner-preferred herbs. It received a safety subtotal of 2.00 ± 0.05 for the two predefined safety items. The prescription-generation efficiency score was 0.86 ± 0.15, with a mean generation time of 9.72 ± 2.11 s, resulting in an overall prescription-consistency score of 7.52 ± 0.44. Compared with the model trained only on practitioner-specific data, the incrementally trained model generated prescriptions more rapidly but showed lower consistency with the practitioner-specific prescribing patterns represented in the expert prescriptions. The manually assessed compatibility consistency item also showed excellent inter-rater agreement in this model, with an ICC(A,2) of 0.953 (95% CI, 0.916–0.980) and a mean inter-rater difference of 0.006 ± 0.102.

Overall, the model trained only on practitioner-specific data more closely reflected the practitioner’s prescribing patterns, whereas the model additionally trained with general TCM knowledge generated prescriptions faster but showed lower consistency. These findings suggest that the balance between practitioner-specific experience and general TCM knowledge should be carefully considered when developing prescription-support models and should not be interpreted as evidence of clinical efficacy or comprehensive clinical safety.

### System deployment and functional realization

In parallel with the development of the core components, DFGLM-TCM was integrated into an operational interactive application system (Fig. 3d). The system frontend provides differentiated functional interfaces and application modes tailored to distinct user roles. The clinician interface supports structured electronic medical record entry, including patient demographics, data from the four diagnostic methods of TCM, and follow-up records, thereby supporting standardized clinical data acquisition and management. It enables clinicians to review complete patient histories, access AI-generated syndrome differentiation results and practitioner-specific prescription references, and query the integrated TCM knowledge base for auxiliary clinical decision support.

For patients, the system implements a QR code-based structured information collection mechanism. Prior to consultation, patients can scan a QR code to complete standardized symptom questionnaires to support data completeness and consultation efficiency. In parallel, an intelligent consultation engine simulates doctor-patient dialogue, guiding patients through multi-round interactions to generate structured diagnostic information that can be used for downstream reasoning and prescription reference.

The student interface focuses on educational applications, providing access to classical TCM texts and academic literature. These resources are integrated with a visualized TCM knowledge graph, enabling students to explore relationships among symptoms, syndromes, treatment principles, and formulas, and to develop a structured understanding of classical medical knowledge.

### Primary-care feasibility and usability results

Following the one-month deployment demonstration at three primary-care hospitals, questionnaire responses were obtained from 35 participating physicians to assess perceived usefulness, workflow compatibility, and user acceptance.

Overall, physicians reported favorable perceptions of the system as a digital clinical support tool (Table 6). A total of 77.14% of respondents agreed that the system could serve as a reference resource for daily clinical practice, while 65.72% considered it compatible with existing primary-care workflows. In terms of knowledge support, 85.72% reported that the knowledge-based question-answering module supported their understanding of TCM theory, and 68.57% indicated that it facilitated interpretation of complex syndrome differentiation. For the experience-oriented component, 71.43% considered the practitioner-specific prescription outputs suitable for use as auxiliary references. These findings represent physicians’ subjective perceptions and do not constitute an objective assessment of prescription quality or clinical effectiveness.

**Table 6:** Attitudes of primary care physicians toward the application of DFGLM-TCM system in routine clinical practice

Item	Agree entirely	Somewhat agree	Undecided	Somewhat disagree	Disagree entirely
Positive					
The platform is helpful as a reference tool for daily clinical practice in primary-care settings	48.57%	28.57%	11.43%	5.71%	5.71%
The platform’s functions are compatible with existing primary-care clinical workflows	34.29%	31.43%	14.29%	14.29%	5.71%
The knowledge retrieval results provided by the platform are clear and easy to understand	40.00%	34.29%	11.43%	8.57%	5.71%
The knowledge-based question answering module supports my understanding of TCM theory during practice	54.29%	31.43%	5.71%	5.71%	2.86%
The practitioner-specific prescription outputs are suitable for use as auxiliary references	28.57%	42.86%	17.14%	5.71%	5.71%
The platform helps me better interpret complex TCM syndromes in routine consultations	37.14%	31.43%	14.29%	11.43%	5.71%
The platform improves my confidence when managing cases that require TCM syndrome differentiation	40.00%	45.71%	8.57%	2.86%	2.86%
Using the platform does not significantly increase my workload during outpatient consultations	28.57%	28.57%	17.14%	14.29%	11.43%
The overall system output is interpretable and suitable for clinical reference rather than direct decision-making	48.57%	42.86%	5.71%	0.00%	2.86%
Overall, the platform is feasible for use as a digital support system in primary-care TCM practice	42.86%	37.14%	11.43%	2.86%	5.71%
I am willing to continue using the platform in future clinical practice	37.14%	45.71%	8.57%	2.86%	5.71%
I would recommend this platform to other primary-care physicians as a clinical reference tool	34.29%	48.57%	8.57%	2.86%	5.71%
Negative/cautious					
The platform requires further optimization before it can be routinely used in primary-care clinics	45.71%	42.86%	5.71%	2.86%	2.86%

Regarding workflow integration, 57.14% of respondents reported that system use did not substantially increase outpatient workload, whereas 42.86% expressed neutral or negative views. This variation suggests that perceived workflow compatibility differed among participating physicians. In addition, 91.43% agreed that the system clearly communicated its role as an auxiliary reference tool rather than a substitute for clinical decision-making.

A total of 80.00% of respondents considered the system feasible as an auxiliary support tool within the evaluated primary-care settings. Furthermore, 82.86% expressed willingness to continue using the system, and the same proportion indicated that they would recommend it to colleagues. However, 88.57% considered further optimization necessary before large-scale routine deployment.

Overall, these findings suggest favorable short-term usability and acceptance of the system in the participating primary-care settings, while further evaluation is needed before broader implementation.

## Discussion

Traditional Chinese Medicine employs a distinctive clinical cognitive system that integrates classical theoretical frameworks with individualized empirical reasoning. Its core concepts, including syndrome differentiation, therapeutic principles, and formula composition, are applied contextually rather than in isolation. These concepts are dynamically adjusted according to the patient’s specific manifestations, the practitioner’s knowledge base, and clinical experience. While foundational theories are documented in classical texts and modern textbooks, expert-level clinical reasoning often remains tacit, accumulated through extensive practice, and is rarely presented in structured form. These features pose inherent challenges for digital representation and computational modeling [40]. Unlike many biomedical fields with clearly defined decision rules, clinical practice in TCM involves flexible pattern recognition, context-sensitive symptom evaluation, and individualized medication selection. Consequently, directly applying LLMs to TCM tasks may conflate general theoretical knowledge with expert-specific reasoning [41], potentially obscuring the distinct roles of these knowledge types in real clinical practice.

In response to these challenges, the present study proposes a task-oriented framework [42] for TCM that explicitly differentiates general domain knowledge from individualized expert experience. The two types of knowledge are modeled through functionally distinct components and integrated within a unified modular system. The principal contribution of DFGLM-TCM lies in this separation, together with component-specific evaluation and preliminary primary-care application, rather than in providing a general-purpose model for autonomous TCM clinical decision-making. This framework addresses the complexity of the TCM knowledge system, the tacit nature of practitioner-specific expertise, and the limited adaptability of general artificial intelligence tools in experience-intensive clinical scenarios.

We assembled a curated TCM knowledge corpus comprising classical texts, modern textbooks, exercise collections, clinical guidelines, and peer-reviewed literature, and organized medical cases extracted from classical texts into a structured knowledge graph. The knowledge-oriented component combined domain adaptation with vector retrieval and retrieval-augmented generation. In the expert-rated evaluation, DFGLM-TCM received the highest descriptive mean score among the evaluated models, while DeepSeek-V3.2 and GPT-4o showed comparable performance. These findings suggest that combining domain-adapted generation with structured knowledge resources may support TCM knowledge retrieval and question answering [43, 44].

Notably, DeepSeek-V3.2 and GPT-4o performed comparably to DFGLM-TCM despite the latter undergoing specialized adaptation using TCM domain knowledge. This finding suggests that domain-specific adaptation may not confer a uniform advantage across all TCM knowledge question-answering tasks, particularly as general-purpose LLMs continue to improve [45]. Nevertheless, high-quality domain-specific resources remain important for knowledge grounding and specialized model development [21], while greater emphasis on expert experience data and specialty-specific datasets may offer a more targeted approach to clinical adaptation. Because the present study did not directly compare the computational efficiency or cost-effectiveness of different adaptation strategies, future studies should evaluate alternative data and training approaches using independent external datasets and standardized measures of robustness, efficiency, and generalizability.

Unlike general knowledge modeling, the experience-oriented component was designed to capture prescribing patterns represented in the clinical records of a senior TCM practitioner. Full-parameter fine-tuning and expert-reviewed augmented cases [46] were used to expand the representation of practitioner-specific diagnostic and therapeutic patterns. Given the inherent variability of TCM prescribing, the evaluation focused on consistency with expert prescriptions rather than exact matching. The experience-oriented component showed a high level of prescription consistency in pulmonary-nodule cases, whereas further fine-tuning with additional general TCM knowledge was associated with lower consistency with practitioner-specific prescribing patterns under the conditions of this comparison. These findings support maintaining a functional distinction between general TCM knowledge and expert-specific experience. However, the evaluation involved only one practitioner and one disease setting and did not include patient follow-up, treatment responses, or objective clinical outcomes. The safety results were limited to the predefined items in the scoring rubric. Thus, the findings provide preliminary evidence that the model can approximate one expert’s prescribing patterns rather than evidence of therapeutic efficacy.

The layered system architecture enabled the coordinated operation of knowledge retrieval, question answering, and practitioner-specific prescription reference through role-oriented interfaces. The one-month primary-care demonstration suggested favorable short-term usability and acceptance among participating physicians, who generally recognized the system as an auxiliary reference tool. Perceptions of workflow compatibility nevertheless varied, and most participants considered further optimization necessary before large-scale routine use. These observations highlight the importance of workflow compatibility, interface design, and appropriate role positioning when introducing AI-assisted tools into primary-care practice.

Several limitations should be acknowledged. First, the knowledge question-answering evaluation was conducted within an expert-curated, textbook-grounded TCM knowledge domain. Because the source textbooks were included in the general TCM corpus, external generalizability to entirely unseen knowledge sources remains to be established. Second, the experience-oriented component was developed primarily using clinical records from a single senior TCM practitioner and was evaluated only in pulmonary-nodule cases. The evaluation focused on prescription-pattern consistency and did not include longitudinal follow-up, treatment-response data, or objective therapeutic outcomes. Therefore, the findings cannot be generalized to other practitioners, diseases, pattern-differentiation systems, institutions, or clinical settings. Third, the primary-care demonstration involved 35 physicians from three institutions over a one-month period and relied primarily on self-reported perceptions. It did not include a control group, long-term follow-up, or objective measures of workflow efficiency, sustained system use, clinician performance, or patient outcomes. The current evidence therefore remains insufficient to support routine or large-scale clinical implementation. Fourth, the prescription-level safety assessment was limited to the predefined checks included in the evaluation rubric, including prohibited herb incompatibilities and the use of toxic herbs. Patient-specific contraindications, dose appropriateness, herb-drug interactions, adverse events, and risks related to comorbidities or concomitant medications were not comprehensively evaluated. Although role-based access control, data anonymization, and physician oversight were incorporated into the evaluated workflow, privacy protection, information security, auditability, and longitudinal system-use monitoring across institutions require further evaluation. System-generated outputs should remain subject to independent review by qualified physicians, who retain responsibility for final clinical decisions.

These limitations indicate several directions for future research. Future studies should incorporate data from multiple practitioners and disease categories, evaluate model performance using independent external datasets, and conduct longer-term multicenter prospective studies with objective clinical and workflow outcomes. Appropriately designed controlled trials may further clarify the effects of AI-assisted prescribing support on long-term patient outcomes [47]. Further system development should also strengthen patient-specific contraindication screening, dose assessment, herb-drug interaction checks, usage logging, post-deployment safety monitoring, and institution-level privacy and governance mechanisms.

## Conclusion

This study developed and evaluated DFGLM-TCM, a modular TCM service system that distinguishes general domain knowledge from expert-specific clinical experience. Through task-oriented training strategies tailored to distinct knowledge and experience functions, together with a unified architecture for multi-task coordination, the system integrates knowledge retrieval, question answering, structured consultation, and practitioner-specific prescription reference within a single service framework. Component-specific evaluations showed favorable performance within the predefined TCM knowledge domain and a high level of consistency with one expert’s prescribing patterns in pulmonary-nodule cases, while the short-term primary-care assessment suggested favorable usability and acceptance.

These findings highlight the potential value of combining differentiated knowledge modeling, task-oriented training, and coordinated multi-task services in the development of TCM-oriented large language model systems. DFGLM-TCM is intended to serve as an auxiliary reference tool under physician oversight, and broader evaluation across practitioners, diseases, institutions, and longer-term clinical settings is needed before wider implementation.

## Supplementary Information


Supplementary Material 1

## Data Availability

The data that support the findings of this study are available from the corresponding author upon reasonable request.

## Abbreviations

AI: Artificial intelligence
LLM: Large language model
TCM: Traditional Chinese Medicine
Q&A: Question answering
RAG: Retrieval-augmented generation
SFT: Supervised fine-tuning
OCR: Optical character recognition
ETL: Extract-transform-load
IRB: Institutional review board
CT: Computed tomography

## Code availability

The underlying code and training/validation datasets are not publicly available but may be made available to qualified researchers upon reasonable request to the corresponding author.
