# Supplementary material for: DFGLM-TCM: an integrated knowledge-and experience-driven large language model system for Traditional Chinese Medicine practice
Source: Chin Med. 2026 Aug 28;21:215. doi: 10.1186/s13020-026-01512-y (PMC13523272; doi:10.1186/s13020-026-01512-y)
Supplement: Supplementary file 1 [file 13020_2026_1512_MOESM1_ESM.pdf]

Table 1: Collection of Knowledge Q&A Questions

Question
1. What are the tongue characteristics and clinical significance of fissured tongue?
2. What are the tongue characteristics and clinical significance of mirror-like tongue?
3. What are the common types of odors in a sickroom?
4. What are the pulse characteristics and clinical significance of weak pulse?
5. What are the pulse characteristics and clinical significance of wiry pulse?
6. What is the Eight-Principle Pattern Identification?
7. What are the clinical manifestations of Qi Deficiency Pattern?
8. What are the clinical manifestations of Blood Stasis Pattern?
9. How to differentiate between Heart Qi Deficiency Pattern and Heart Yang Deficiency Pattern?
10. What are the clinical manifestations of Spleen Qi Sinking Pattern?
11. What is the Six-Meridian Pattern Identification?
12. What are the clinical manifestations of Deficiency-Heat in Blood Aspect Pattern?
13. What is the concept of "Pattern" (Zheng) in TCM?
14. How are the Yin-Yang attributes of things classified?
15. What are the generating and controlling sequences of the Five Phases?
16. What are the physiological functions of the Spleen?
17. What are the physiological functions and characteristics of the Gallbladder?
18. What is the relationship between the Lung and the Liver in TCM?
19. What are the sources of Qi in the human body?
20. What is the fundamental concept of Blood in TCM?
21. How to understand the statement "Blood is the mother of Qi"?
22. What is the narrow definition of Essence (Jing)?
23. What is the basic concept of meridians?
24. What are the directional and connecting rules of the Twelve Regular Meridians?
25. Which meridian is known as the "Sea of the Twelve Meridians"?
26. What is the concept of constitution in TCM?
27. What are the characteristics of the Metal-Type constitution?
28. What are the pathogenic characteristics of Pestilential Qi?
29. What are the Six Excesses (Six Pathogenic Factors)?
30. What are the etiology and pathogenesis of Phlegm-Retention?
31. How to differentiate between Deficiency-type and Excess-type wheezing?
32. What are the key points for pattern identification in diarrhea?
33. How are the Liver and Spleen related to the onset of stomach pain?
34. What are the key points for pattern identification in dysentery?
35. What are the key points for pattern identification and treatment principles for abdominal mass (Ji-Ju)?
36. What are the causes and treatment principles for edema?
37. What are the common patterns, treatment methods, and representative formulas for palpitations?
38. What are the treatment principles for chest impediment (chest pain)?
39. How to differentiate between depressive psychosis (Dian) and dementia?
40. How to differentiate the nature of abdominal pain?
41. What are the Eight Treatment Methods in TCM?
42. What is herbal compatibility (formulation)?
43. What is a powdered preparation (San)?
44. What are the functions, indications, and key points for pattern identification of Ephedra Decoction (Ma Huang Tang)?
45. What are the functions, indications, and key points for pattern identification of Cinnamon Twig Decoction (Gui Zhi Tang)?
46. What are the functions, indications, and key points for pattern identification of Lonicera and Forsythia Powder (Yin Qiao San)?
47. What is the composition and usage of Major Purgative Decoction (Da Cheng Qi Tang)?
48. What precautions should be taken when using purgative formulas?
49. What is the composition and usage of Hemp Seed Pill (Ma Zi Ren Wan)?
50. What are harmonizing formulas (He Jie)?
51. What are the categories of heat-clearing formulas?
52. What are the key points for pattern identification of Rhinoceros Horn and Rehmannia Decoction (Xi Jiao Di Huang Tang)?
53. What precautions should be taken when using Universal Benefit Toxin-Dispersing Beverage (Pu Ji Xiao Du Yin)?
54. What does "All things originate, and the Five Movements cycle through the heavens" mean?
55. How does the "Inner Canon" explain the universe and all things from a monistic perspective?
56. What does "The laws of the four seasons complete all things" mean?
57. What is the correspondence between the Five Tones and the Five Phases?
58. What is the concept of Zang-Fu manifestation (visceral manifestation)?
59. What does "The stomach is full while the intestines are empty" mean?
60. How does the "Inner Canon" describe the concept of Nutritive and Defensive Qi?
61. What are the possible outcomes of Taiyang Disease?
62. How is Cinnamon Twig Decoction (Gui Zhi Tang) interpreted in the "Treatise on Cold Damage"?
63. How to differentiate between Taiyang Cold Damage and Taiyang Wind Strike?

64. What does "Water Qi below the heart" mean?
65. How to understand "Damaged Disease" (Huai Bing) in the "Treatise on Cold Damage"?
66. How is Gardenia and Fermented Soybean Decoction (Zhi Zi Chi Tang) interpreted in the "Treatise on Cold Damage"?
67. What are the characteristics of Warm Diseases?
68. What is Wind-Heat Pathogenic Factor?
69. What is Warm-Heat Pathogenic Factor?
70. What is Defensive Aspect Pattern?
71. How to perform pattern identification for bleeding gums in Warm Diseases?
72. What does wiry pulse typically indicate in Warm Diseases?
73. What are the main treatment methods for Warm Diseases?
74. What are the diagnostic criteria for Wind-Warmth Disease?
75. What are the similarities and differences between Ephedra Plus Atractylodes Decoction Syndrome and Ephedra, Apricot Kernel, Coix, and Licorice Decoction Syndrome?
76. How to understand "All vessels share one origin, and all disorders stem from it"?
77. What is Wind Stroke (Zhong Feng)?
78. What are the main symptoms, pathogenesis, treatment method, and herbal characteristics of Cinnamon Twig, Peony, and Anemarrhena Decoction Syndrome?
79. Both Minor Constructive Decoction and Cinnamon Twig Plus Dragon Bone and Oyster Shell Decoction treat dual deficiency of Yin and Yang in consumptive diseases. What are their similarities and differences?
80. Based on the original text, what are the etiology, pathogenesis, and basic treatment principles for chest impediment and heart pain?
81. Both Trichosanthes, Allium, and Pinellia Decoction and Aconite and Red Halloysite Pill treat "pain radiating through the back." What are their differences?
82. What are the key points for differentiating deficiency and excess in abdominal fullness?
83. What is the concept and clinical manifestations of acute mastitis (Ru Yong)?
84. What are the clinical characteristics of headed carbuncle (You Tou Ju)?
85. What are the concept, clinical characteristics, TCM patterns, and representative formulas for erysipelas (Dan Du)?
86. How many stages are there for internal hemorrhoids, and what are their clinical characteristics?
87. What is the anatomical and clinical significance of the dentate line?
88. What are the treatment principles for metrorrhagia and metrostaxis (Beng Lou)?
89. What are the key points for pattern identification in dysmenorrhea?
90. What are the medication principles during pregnancy?
91. What are the concepts of pregnancy edema (Zi Zhong), pregnancy dizziness (Zi Yun), and eclampsia (Zi Xian)?
92. What are the key points for pattern identification in persistent lochia (E Lu Bu Jue)?
93. What are the main achievements and influences of the "Yellow Emperor's Inner Canon"?
94. What are the main achievements of the "Compendium of Materia Medica" (Ben Cao Gang Mu)?
95. Who are the Four Great Schools of the Jin-Yuan period, and what are their main academic viewpoints?
96. What are the main achievements of "Essential Prescriptions Worth a Thousand Gold" (Bei Ji Qian Jin Yao Fang) and "Supplement to the Essential Prescriptions" (Qian Jin Yi Fang)?
97. Discuss Liu Wansu's methods for treating fire-heat diseases.
98. How does the "Danxi's Mastery of Medicine" describe the etiology and pathogenesis of depression patterns?
99. Briefly explain Yang Jizhou's statement "Acupuncture and herbal medicine are both indispensable."
100. How many types of leukorrhagia (Dai Xia) did Fu Shan classify?

Table 2 Comprehensive Scoring Table for Simulated Prescriptions

Rating Category	Grading Criteria	Score
Similarity		7 points
prescription size	The number of herbals falls within the commonly used range in expert prescriptions.	1 point
Specialized medication for specific diseases	The prescription contains characteristic drugs for pulmonary nodules commonly used by experts.	1 point
Compatibility consistency	Compared with expert prescriptions: 3.5 points for having the same 5 flavors or more of different types of traditional Chinese medicine; 3 points for having similar 5 flavors or more of different types of traditional Chinese medicine; 2.5 points for having the same four flavors of different types of traditional Chinese medicine; 2 points for having similar four flavors of different types of traditional Chinese medicine; 1.5 points for having the same three flavors of different types of traditional Chinese medicine; 1 point for having similar 3 flavors of different types of traditional Chinese medicine.	3.5 points
habitual medication	The proportion of commonly used traditional Chinese medicines that AI prescription covers, according to experts' habits: <70%: 0 points; 70% to <80%: 0.5 points; 80% to 90%: 1 point; >90%: 1.5 points.	1.5 points
Safety		2 points
incompatibility of drugs in a prescription	The prescription does not contain any incompatibility, or although it contains incompatibility, it conforms to the commonly used compatibility habits of experts.	1 point
Use of toxic drugs	The prescription does not contain excessive use of toxic traditional Chinese medicine, or even if there is excessive use, it does not exceed the commonly used dosage by experts.	1 point
Prescription output speed	For AI prescription output speed, 1 point will be given for completion ≤10 seconds: 1 point; >10 to ≤15 seconds: 0.7 points; >15 to ≤20 seconds: 0.5 points; >20 to ≤30 seconds: 0.3 points; >30 seconds: 0 points.	1 point

Table 3 Baseline characteristics of patients with pulmonary nodules enrolled in the study

Variable	N (%)	Range	Mean ± SD	Median (IQR)
Gender				
Male	200 (44.05%)			
Female	254 (55.95%)			
Age		23-90	52.18 ± 15.08	53 (39-62）
Maximum diameter of nodule（mm）		2-30	6.8 ± 4.12	6 (5-7)
Lung-RADS classification				
Classes 2	209 (46.04%)			
Classes 3	105 (23.13%)			
Classes 4A	49 (10.79%)			
Classes 4B	69 (15.20%)			
Classes 4X	17 (3.74%)			
Unknown	5 (1.10%)			
